# Hypertension guideline implementation and blood pressure control in Matlosana, South Africa

**DOI:** 10.4102/safp.v66i1.5964

**Published:** 2024-10-30

**Authors:** Keolebile I. Ditlhabolo, Carien Lion-Cachet, Ebrahim Variava

**Affiliations:** 1 Department of Family Medicine and Primary Health Care, Faculty of Health Sciences, University of the Witwatersrand, Johannesburg, South Africa; 2 Department of Family Medicine, Primary Health Care Matlosana Sub-district, Dr Kenneth Kaunda District, North West Department of Health, Klerksdorp, South Africa; 3 Department of Internal Medicine, Faculty of Health Sciences, University of the Witwatersrand, Johannesburg, South Africa; 4 Department of Internal Medicine, Tshepong Hospital, Dr Kenneth Kaunda District, North West Department of Health, Klerksdorp, South Africa

**Keywords:** hypertension, management, guidelines, blood pressure control, control targets, implementation, primary care, comorbidities, target organ damage

## Abstract

**Background:**

High systolic blood pressure remains a leading modifiable risk factor for cardiovascular diseases worldwide and in South Africa (SA). Information about the extent of guideline implementation and blood pressure (BP) control is lacking in Matlosana Sub-district, North West province, SA. The study aimed to assess the implementation of the South African Hypertension Practice Guideline (SAHPG) and BP control in adults attending primary care facilities in Matlosana.

**Methods:**

Cross-sectional study was conducted, using 523 randomly sampled medical records. Data collected included demographic information, recorded BP readings, anthropometry, screening for target organ damage (TOD), hypertension complications, comorbidities, lifestyle advice and drug therapy.

**Results:**

According to the reviewed records the mean age of the participants was 56.77 years with a standard deviation of 12.4 years and 376 (71.9%) records belonged to females. Blood pressure control was documented in 229 (43.8%) of the medical records, with better control recorded in a group with comorbid human immunodeficiency virus (HIV) than in groups with other comorbidities.

**Conclusion:**

The study found poor documentation of the SAHPG recommendations among patients with hypertension. According to the patient records BP control was suboptimal, the most common documented comorbid illness was HIV, and screening for TOD was generally poorly documented.

**Contribution:**

Programmes that audit and improve the quality of hypertension guideline implementation and BP control in primary care require ongoing support and research.

## Introduction

Globally, high systolic blood pressure (SBP) remains a leading modifiable risk factor for premature cardiovascular deaths, accounting for 10.8 million (95% confidence interval [CI]: 9.15–12.1 million) cardiovascular deaths and 11.3 million (95% CI: 9.59–12.7 million) deaths in 2021.^1^ An estimated 874 million people worldwide had an SBP of 140 mmHg or more in 2015, with this figure projected to increase.^2^ According to the World Health Organization (WHO), an estimated 1.28 billion adults aged 30–79 years worldwide have hypertension, with two-thirds living in low- and middle-income countries.^3^ The highest prevalence of hypertension was observed in sub-Saharan African countries, accounting for two-thirds of the world’s burden of disease.^4^ South Africa (SA) among other sub-Saharan African countries has experienced a surge in cardiovascular disease (CVD) burden and its associated risk factors including hypertension.^5^ According to the WHO four of every five people with hypertension are not adequately treated.^6^ The highest prevalence of uncontrolled hypertension (≥ 140/90 mmHg) is reported in sub-Saharan African countries, where treatment for hypertension is unacceptably low.^7^ In SA, BP control varies, and the prevalence of uncontrolled hypertension ranges from 13.5% to 75.5%,^8,9^ with variations attributed to sociodemographic and clinical factors impacting the control of hypertension.^9^

The United Nations Sustainable Development Goals (SDGs) call for 80% control rates by 2030, highlighting the urgent need for improvement in hypertension control.^10^ The 2021 WHO guideline on the pharmacological treatment of hypertension recommends achieving optimal BP targets in order to reach the SDGs.^11^ The main goal of BP control is to reduce hypertension target organ damage (TOD).^11^ There are various clinical practice guidelines (CPGs) available nationally and internationally to assist healthcare practitioners manage hypertension.^11,12,13,14^ The CPGs are evidence-based documents developed to reduce disparities in clinical practice.^15^ They are defined as a set of recommendations generated from systematic reviews of current clinical evidence, assessment of risks and benefits of options available for the diagnosis and treatment of diseases.^15^ The role of CPGs is to standardise medical care, by ensuring that patients receive the same treatment regardless of their geographical location or clinician expertise, thereby improving the quality of healthcare.^15^

In primary health care (PHC) in SA there are standard treatment guidelines (STGs) that guide the diagnosis, monitoring, drug therapy, screening for cardiovascular risks, lifestyle modification and screening for TOD according to the particular CPG.^12^ The South African hypertension practice guideline (SAHPG), formulated for the management of hypertension in this country, has set the BP control target to a BP reading of less than 140/90 mmHg regardless of the cardiovascular risk, in order to simplify hypertension management.^13^ The SAHPG has also set out recommendations regarding TOD to screen for hypertensive patients, routine investigations, lifestyle modification, antihypertensive therapies and the compelling indications for these antihypertensive therapies.^13^ As in SA, most international guidelines recommend lifestyle modification before antihypertensive therapy.^11,14^ Lifestyle change has been shown to reduce BP by as much as 3.5 mmHg by maintaining a healthy lifestyle, thereby reducing cardiovascular risks by approximately 30% before adding antihypertensive therapy to the treatment.^16^ Lifestyle modification includes reducing salt intake, reducing weight if overweight or obese, physical activity, smoking cessation and reducing alcohol intake.^13^ The commonly used classes of antihypertensive therapies are diuretics, calcium channel blockers (CCB), angiotensin-converting enzyme inhibitors (ACEI), angiotensin receptor blockers, alpha-blockers, beta-blockers and vasodilators.^13^

There is also an ideal clinic framework that emphasises integrated clinical service management and supports the availability of the guidelines in each consultation room.^17^ Ideal clinic is a framework that sets out the standards to which PHC facilities must align in order to ensure the provision of good-quality health services.^17^ This is achieved by ensuring that the clinic has good infrastructure, adequate staff members, medicines, supplies and bulk supplies, as well as sound administrative processes. According to the ideal clinic checklist of elements, at least 80% of the clinic records audited for the priority health conditions, including hypertension and diabetes, should be guideline compliant.^17^

The STGs and essential medicines list for SA is available on the Knowledge Hub website as a PDF document, which can be downloaded to a cellular phone for convenience of use whenever needed.^11^ The Knowledge Hub is an electronic learning platform, hosted by the South African Department of Health, that provides access to personalised professional development opportunities and resources including online and blended learning courses, face-to-face workshops, guidelines and many other resources.^18^ The SAHPG is also accessible from the Internet for the comprehensive management of hypertension.^13^ Previous studies showed that the target for the management of hypertension is barely achieved,^19,20^ because of poor uptake of CPG recommendations.^21^ Similarly, in another study poor CPG uptake was attributed to a lack of familiarity with the guideline, individual perception about the guideline, a lack of access to the guideline itself and resistance to change.^22^

Blood pressure (BP) control varies widely in SA settings,^19,20^ and information about the extent of guideline implementation and BP control is lacking in the Matlosana Sub-district. This study aims to determine the extent of SAHPG implementation and BP control achieved in adults with hypertension attending primary healthcare facilities in the Matlosana Sub-district. The association between BP control with demographic factors, comorbidities, TOD, antihypertensive therapy prescribed and lifestyle advice provided in Matlosana Sub-district primary health care facilities were explored.

## Research methods and design

### Study design

A cross-sectional study was conducted using data collected from the active medical records of patients consulting at four primary care clinics.

### Study setting

**FIGURE 1:**
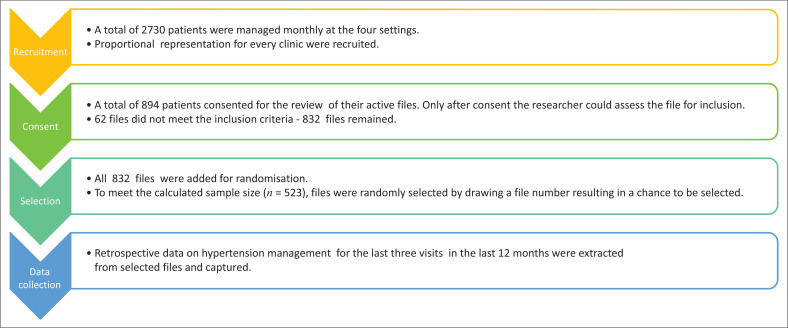
Flow diagram illustrating the sequential process from population sampling and recruitment to obtaining consent, file selection and data extraction.

The study was conducted in the Matlosana Sub-district, one of the four sub-districts in Dr Kenneth Kaunda District, situated southwest of Johannesburg, SA. Based on the population census, Matlosana Sub-district had a population of 431 231 people in 2017.^23^ There are 18 PHC facilities in the sub-district, comprising four community health centres (CHCs) and 14 PHC day-clinics that operate between 07:00 and 16:00. All facilities in the sub-district use paper-based medical records. According to the CHC registers and daily hypertension medical record counts taken by the researcher in August 2020, there were an estimated 2730 medical records of hypertensive patients seen at the four CHCs monthly (Figure 1). All the CHCs are situated in different townships in the sub-district, with each CHC supporting the PHC day-clinics in the same demarcation area for services not available in the day-clinics such as electrocardiogram (ECG), ultrasound services, doctors’ availability in the CHCs every day and after 16:00 h coverage. All CHCs operated 24 h a day, had full-time nurses for 24 h and daily doctors’ coverage during the week until 16:00. At the time of the study not all day-clinics had daily doctor coverage because of the shortage of doctors in the sub-district; some had a doctor twice a week and others three times a week. The CHCs provided a full range of services including antenatal care, women’s and children’s health, mental health, human immunodeficiency virus (HIV)/acquired immunodeficiency syndrome (AIDS), sexually transmitted infections (STIs) and tuberculosis (TB) (HAST) programme, adolescent and youth friendly services, acute care, chronic care, as well as the promotive and preventive services. The study was conducted in all four CHCs in the sub-district to obtain a representative sample and generate findings that are generalisable to the sub-district.

### The study population and sampling strategy

The target population were all adult patients living with hypertension who were followed up in the four CHCs in Matlosana Sub-district. The inclusion criteria used included: hypertensive patients 18 years and above who consented to the utilisation of their medical records for the study; who had hypertension follow-up visits at the same facility and documented in the same paper-based medical record because of the unavailability of electronic medical records; who had more than two hypertension follow-up visits in the past year to assess BP control at the first two visits and compare BP control at the third visit; and the patients had to be on at least one recorded prescribed antihypertensive treatment to be eligible for the study. The exclusion criteria used included: documented pregnancy with the utilisation of a different hypertension guideline during pregnancy; antenatal care follow-up duration of less than a year; pregnant patients’ medical records or medical records of hypertensive patients after 16:00 because of the shortage of staff at night (with only two professional nurses and one assistant or enrolled nurse on duty at night this resulted in no assistance with obtaining the patient’s informed consent); and patients who needed emergency care management.

According to the CHC daily hypertension medical record counts performed by the researcher in August 2020 at the four facilities, there were an estimated 2730 medical records of hypertensive patients seen at the four CHCs monthly (Table 1). A minimum sample size was calculated using the formula^24^:


$$n=\frac{N}{1+N{\left(e\right)}^{2}}$$ [Eqn 1]

where *n* is the sample size, *N* is the population size estimated at 2730, and assuming a margin of error of 0.05, a sample size of 345 was calculated at a confidence interval of 95%. This number was increased by 50% to account for missing data from the medical records, which enlarged the sample size to 523. To ensure that all the facilities in the study were well represented, the sample size calculated was distributed according to the estimated proportion of hypertensive patients counted at each of the facilities (Table 1). Patients were conveniently recruited during their clinic visits and all the medical records of patients who signed the consent forms were identified to be screened for inclusion in the study. All the medical records that met the inclusion criteria were selected using random sampling without replacement (Figure 1).

**TABLE 1:** Population size, sample proportion and sample size number at each of the four community health centres.

CHC	Population size (per facility)	Sample proportion (%)	Calculated sample (size per facility)
GM-CHC	1040	38.1	199
J-CHC	884	32.4	170
T-CHC	416	15.2	79
B-CHC	390	14.3	75

**Total**	**2730**	**100.0**	**523**

B, Botshabelo; CHC, community health centre; GM, Grace Mokhomo; J, Jouberton; T, Tigane.

### Pilot study

A pilot study was conducted using 40 participants to determine the feasibility and suitability of the methodology to achieve the objectives of the study. Informed consent to conduct the pilot study was obtained from the participants. There were no adjustments required to the measurement tool after the pilot study because the measuring tool was feasible. The medical records reviewed during the pilot study were not included in the main study because the registry clerk and clinicians were not yet familiar with the process.

### Measurement tool and data collection

The researcher developed a measurement tool using the SAHPG.^13^ The measurement tool had five parts. The first part of the measurement tool comprised the demographic information of participants, which included age, sex and employment status. Age affects the course and progression of a disease and is important in determining the correct course of treatment.^25,26^ In this study age was categorised into three groups (< 50 years, 50–60 years, > 60 years). The second part of the tool assessed the implementation of the hypertension guideline. The guideline elements included were anthropometry (weight, height and body mass index [BMI]), BP measurements at the last three follow-up visits, documented urine dipstick test at least once a year, documented random blood glucose at least once a year if nondiabetic, creatinine and estimated glomerular filtration rate at least once a year, and random cholesterol once a year. The BP measurements at the last three consecutive follow-up visits were collected from the medical records. Blood pressure control was categorised into three groups: controlled if BP was < 140/90 mmHg at all three visits; equivocal control if BP was < 140/90 mmHg at either one or two follow-up visits and uncontrolled if BP was ≥ 140/90 mmHg at all three visits. In this study, the BP control target was defined as a BP < 140/90 mmHg with a diastolic blood pressure (DBP) of < 90 mmHg and a SBP of < 140 mmHg.^13^ The BP reading at visits 2 and 3 were used to assess BP control, where BP was controlled if the BP reading at both visits 2 and 3 were < 140/90 mmHg, and uncontrolled if BP was ≥ 140/90 mmHg at either visit 2 or 3 or both visits. Blood pressure control at the last two visits (visits 2 and 3) was further compared to BP control achieved when all three visits (visits 1, 2 and 3) were used to assess BP control.

The BMI was defined as a person’s weight in kilograms divided by the square of their height in metres.^27^ The BMI was also collected under anthropometry to assess guideline implementation and the association between BP control and BMI. The BMI was categorised into underweight if < 18.5 kg/m^2^, normal if ranging from 18.5 kg/m^2^ to 24.9 kg/m^2^, overweight if ranging from 25 kg/m^2^ to 29.9 kg/m^2^ and obese if > 30 kg/m^2^.^27^

The third part of the guideline assessed for the presence of comorbidities (diabetes mellitus, dyslipidaemia, HIV, gout and asthma) and the screening of TOD (ischaemic heart disease, heart failure and kidney disease). The fourth part of the tool assessed lifestyle advice and the type of lifestyle advice given. The fifth part of the measurement tool assessed the drug therapy prescribed and the total number of drug therapies prescribed. During the recruitment of participants to screen for the eligibility criteria, two of the facilities were visited in the morning between 07:00 and 09:00 and the other two facilities were visited in the afternoon between 14:00 and 16:00. The researcher alternated morning and afternoon visits between the facilities for 5 days a week. The researcher informed the patients who were sitting in the waiting area about the study, and an information sheet was given to each patient to read. Patients were informed that participation in the study was voluntary and that their refusal would not affect the care they were going to receive. Those who were interested then signed a consent form for the utilisation of their medical records with the clinicians at each of the CHCs at the end of the consultation. There were no recent pre-existing registers of hypertensive patients in the CHCs, therefore, medical records that met the inclusion criteria for the study were kept separate for the researcher to create a single register list, and thereafter, the records were immediately returned to the records room. Participants were recruited from 01 August 2022 to 06 October 2022. Medical records were randomly selected from the list using simple random sampling without replacement.^28^ Medical record numbers were marked on paper according to the register list, then folded and placed in a bowl. The numbers were blindly stirred after each selection was made and the corresponding medical record was identified until 523 medical records were selected for sampling. Each medical record stood an equal chance to be selected once. The registry clerks, who were not part of the study, were requested to retrieve the selected medical records using the patient medical record numbers. These numbers were provided to them by the researcher. The medical records were immediately returned to the clerks for back-filing after data collection. Data on the dependent variables (BP control assessed by collecting BP recorded at the last three consecutive visits) and independent variables (demographic information, anthropometry, documented comorbidities and TOD, screening for TOD and antihypertensive therapy) were collected. The register list of medical record numbers was destroyed by using a shredding machine after the data collection.

### Data analysis

The manually collected data were captured in Microsoft Excel and analysed using STATA version 16 (Stata Corp, College Station, Texas, United States).^29^ The researcher used descriptive statistics to describe the categorical and continuous variables. Categorical variables were summarised using frequencies and percentages. Continuous variables were summarised as median and interquartile range if the data were non-normal or mean and standard deviation if the data were normally distributed. The categorical variables were sex, employment status, BMI, documented urine dipstick test, documented urine protein, documented blood glucose test, documented random cholesterol and ECG performed. The continuous variables were age, recorded BP reading, number of antihypertensive therapies prescribed and number of documented comorbidities. A histogram plot was used to assess normality. A bivariate analysis was used to assess the correlation between the BP control status, demographic and clinical factors. To determine the factors associated with BP control, a logistic regression was performed, and odds ratios (ORs) were calculated. A *p*-value < 5% was regarded as statistically significant.

### Ethical considerations

Permission to conduct the study was obtained from the Human Medical Ethics Committee University of the Witwatersrand (M2111116) and written permission was obtained from the North West Department of Health Research Monitoring and Evaluation Directorate and Dr Kenneth Kaunda District Research Committee. Because of the utilisation of active files, informed consent was obtained from all the participants before the study. The patients’ confidentiality and anonymity were ensured by keeping the data collection sheets anonymous and by deleting any identifiers that linked the patients’ medical records to the questionnaire. The data collection sheets will be securely stored for 2 years after publication of the results, after which they will be discarded by using a shredding machine. The electronic data were anonymised and stored under password protection and will be deleted 2 years after the publication of the study.

## Results

### Documented demographic characteristics

Table 2 presents the sociodemographic information extracted from the review of the documented medical records. The majority of the participants were females 376 (71.9%). Among the three age groups, the bigger group was 214 (40.9%) of those who were older than 60 years.

**TABLE 2:** Patients’ demographic characteristics (*N* = 523) extracted from the medical records.

Category	Frequency		BP controlled		BP uncontrolled		OR	95% CI	*p*
	*n*	%	*n*	%	*n*	%			
**Age (years)**									
< 50	142	27.15	60	42.25	82	57.75	-	-	-
50–60	167	31.93	81	48.50	86	51.50	1.29	0.82–2.02	0.118
> 60	214	40.92	88	41.12	126	58.88	1.21	0.53–2.78	0.653
**Sex**									
Unspecified	1	0.19	1	0.19	0	0.00	-	-	-
Male	146	27.97	57	39.04	89	60.56	-	-	-
Female	376	72.03	171	45.48	205	54.52	1.31	0.88–1.92	0.177
**Employment status**									
Unemployed	257	49.14	111	43.19	146	56.81	-	-	-
Employed	33	6.31	20	60.61	13	39.39	2.02	0.62–4.24	0.062
Not specified	233	44.55	98	42.06	135	57.94	0.95	0.67–1.37	0.800

BP, blood pressure; OR, odds ratio; 95% CI, confidence interval.

### Documented blood pressure and body mass index

The results in Table 3 showed that the achievement of BP control at visits 2 and 3 was documented in 229 (43.8%) medical records (Table 2). However, when including visit 1, with BP control assessed for all three visits, there was a drop in BP control achieved in 126 (24.09%) medical records. Furthermore, it was found that BMI was not recorded in 284 (54.3%) of the medical records.

**TABLE 3:** Record review of documented blood pressure levels and body mass index (*N* = 523).

Variable	Category of BP	Controlled		Uncontrolled		Control equivocal	
		*n*	%	*n*	%	*n*	%
Summary of BP control (last 2 visits)	Visit 2	229	43.79	294	56.21	-	-
	Visit 3	229	43.79	294	56.21	-	-
Summary of BP control (3 visits)	Recorded BP	126	24.09	161	30.78	236	45.12
Recorded BMI in 3 groups according to BP control	Not specified (*n* = 284)	80	28.17	84	29.58	120	42.25
	Underweight (*n* = 8)	7	87.50	0	0.00	1	12.50
	Normal (*n* = 69)	23	33.82	11	16.18	35	50.72
	Overweight (*n* = 69)	22	31.15	16	18.03	31	50.82
	Obese (*n* = 93)	18	19.57	25	26.88	50	54.35

BP, blood pressure; BMI, body mass index.

### Recorded hypertension comorbidities, target organ damage and blood pressure control

The results in Table 4 describe the documented screening tests performed for TOD, hypertension comorbidities and BP control. From the 523 medical records reviewed, data were collected for documented urine analysis, urine protein, random blood glucose, random cholesterol, serum creatinine, serum potassium level and ECG. No association was found between BP control and the screening for TOD. There were 341 (65.2%) medical records with documented comorbid conditions that included diabetes mellitus, dyslipidaemia, HIV, asthma and gout, while the recorded TOD included heart failure, stroke and ischaemic heart disease. The three most common comorbidities found were HIV 218 (41.7%) followed by dyslipidaemia 133 (25.4%) and diabetes mellitus 85 (16.3%). There were 18 (3.44%) medical records with documented asthma and 2 (0.38%) medical records with documented gout comorbidities. Furthermore, comorbidities overlapped between the groups. In the dyslipidaemia group, there were 59 (44.4%) medical records with documented comorbid diabetes mellitus and 35 (16.1%) with documented comorbid HIV. In the diabetes mellitus group, there were 20 (23.5%) medical records with documented comorbid HIV and 59 (69.4%) with comorbid dyslipidaemia. Diabetes mellitus often existed with other comorbidities as documented in 79 (92.9%) records. Lastly, the patients’ files in the comorbid HIV group had 1.59 times (odds ratio [OR] = 1.59; 95% CI: 1.12–2.26; *p* = 0.009) increased odds of having better documented BP control than the patients’ files with no comorbid HIV.

**TABLE 4:** Recorded target organ damage screening, hypertension comorbidities and blood pressure control (*N* = 523).

Variable	Category	Frequency (*n* = 523)		BP controlled		BP uncontrolled		Odds ratios	95% CI	*p*
		*n*	%	*n*	%	*n*	%			
Screening carried out	Urine dipstick	120	22.90	56	25.50	64	21.80	0.14	0.8–1.64	0.469
	Urine protein	92	17.60	44	47.80	48	16.30	0.82	0.52–1.29	0.390
	Random blood glucose	245	46.80	107	43.80	138	47.20	0.99	0.7–1.4	0.961
	Creatinine/eGFR	398	76.10	182	79.50	216	73.50	1.19	0.97–1.45	0.067
	Potassium	126	24.09	59	25.80	67	22.80	0.85	0.57–1.27	0.430
	Random cholesterol	399	76.30	183	79.90	216	73.50	1.44	0.95–2.17	0.086
	ECG	12	2.30	7	3.10	5	1.70	0.55	0.17–1.75	0.304
Recorded comorbidities	Diabetes mellitus	85	16.30	34	40.00	51	60.00	0.61	0.21–1.73	0.442
	Dyslipidaemia	133	25.40	51	38.35	82	61.65	0.56	0.19–1.64	0.144
	Ischaemic heart disease	1	0.19	0	0.00	1	100.00	-	-	-
	Heart failure	4	0.76	4	100.00	0	0.00	-	-	-
	HIV	218	41.70	110	50.46	108	49.54	1.42	0.98–2.05	0.009
	Gout	2	0.38	1	50.00	1	50.00	-	-	-
	Asthma	18	3.44	11	61.11	7	38.89	-	-	0.997
	Stroke	1	0.19	0	0.00	1	100.00	-	-	-
Number of medical records with recorded comorbidities	0 HPT comorbidity	182	-	75	41.21	107	58.79	-	-	-
	1 HPT comorbidity	243	-	111	45.68	132	54.32	-	-	0.390
	2 HPT comorbidities	79	-	34	43.04	45	56.96	-	-	0.360
	3 HPT comorbidities	19	-	9	47.37	10	52.63	-	-	0.780

HPT, hypertension; BP, blood pressure; eGFR, estimated glomerular filtration rate; ECG, electrocardiogram; HIV, human immunodeficiency virus; 95% CI, 95% confidence interval.

### Recorded monitoring and screening for target organ damage

Table 5 shows the documented proportion of screening tests performed for TOD. These recorded screening tests were compared between six groups including the HIV, dyslipidaemia and diabetes mellitus groups. It was found that the screening tests performed included urine analysis for proteinuria, serum creatinine and glomerular filtration rate, serum potassium, random cholesterol and glucose, and ECG tracings. In addition, urine testing for protein and blood glucose was performed in the diabetes mellitus and dyslipidaemia groups using a statistically significant frequency. It was also discovered that serum creatinine and total cholesterol testing were performed significantly more often in the comorbid HIV group. Furthermore, it was determined that screening tests for TOD in patients with documented comorbid HIV were recorded at a statistically significant frequency than in patients with no comorbid HIV.

**TABLE 5:** Recorded screening for target organ damage in relation to documented comorbidities (*N* = 523).

Category of screening recorded	TOD – Screening frequency (*n* = 523)		Comorbidities																	
			Diabetes mellitus (*n* = 85)			Dyslipidaemia (*n* = 133)			HIV (*n* = 218)			Asthma (*n* = 18)			Gout (*n* = 2)			IHD (*n* = 1)		
	*n*	%	*n*	%	*p*	*n*	%	*p*	*n*	%	*p*	*n*	%	*p*	*n*	%	*p*	*n*	%	*p*
Urine dipstick	120	22.94	37	43.53	< 0.001*	47	35.34	< 0.001*	50	22.94	0.997	7	38.89	0.102	2	100.0	0.362	1	100.0	0.585
Urine protein	92	17.59	32	37.64	< 0.001*	36	27.07	< 0.001*	37	16.97	0.754	7	38.89	0.016*	0	0.0	0.513	0	0.0	0.644
Creatinine/eGFR	398	76.01	68	80.00	0.357	109	81.96	0.067	217	99.54	< 0.001*	15	83.33	0.464	2	100.0	0.427	1	100.0	0.074
Potassium	126	24.09	45	52.94	< 0.001*	49	36.84	< 0.001*	55	25.23	0.607	7	38.89	0.135	1	50.0	0.391	0	0.0	0.573
Random cholesterol	399	76.29	68	80.00	0.549	75	56.39	0.123	218	100.00	< 0.001*	16	88.89	0.201	2	100.0	0.431	1	100.0	0.073
Random blood glucose	245	46.85	62	72.94	< 0.001*	108	81.20	0.011*	109	50.00	0.222	11	61.11	0.201	2	100.0	0.131	0	0.0	0.317
ECG	12	2.29	4	4.71	0.105	5	3.76	0.237	7	3.21	0.191	1	5.56	0.347	0	0.0	0.828	0	0.0	0.828

TOD, target organ damage; HIV, human immunodeficiency virus; IHD, ischaemic heart disease; eGFR, estimated glomerular filtration rate; ECG, electrocardiogram.

* , *p* < 0.05.

### Antihypertensive therapy prescribed and lifestyle advice in reviewed medical records

The results in Table 6 showed that lifestyle advice was documented in 521 (99.2%) of the medical records. In addition, all the medical records had at least one documented prescription of antihypertensive therapy; 124 (23.7%) medical records had a single documented antihypertensive therapy prescribed and 399 (76.29%) medical records had more than one documented antihypertensive therapy prescribed. An increase in the number of antihypertensives prescribed by one drug reduced the odds of having controlled BP by 25% (OR = 0.75; 95% CI: 0.61–0.94). Patients on enalapril medication were 39% less likely of having controlled BP (OR = 0.61; 95% CI: 0.43–0.88) compared to those not on enalapril. Patients on drug combinations were 52% less likely to have controlled BP (OR = 0.48; 95% CI: 0.33–0.74) compared to patients on a single antihypertensive. In the adjusted regression model, patients on antihypertensive combinations had 47% reduced odds of having controlled BP (adjusted odds ratio [AOR] = 0.53; 95% CI: 0.45–0.1) compared to those on a single antihypertensive therapy.

**TABLE 6:** Drug therapy and lifestyle advice documented in patient records (*N* = 523).

Prescribed treatment	Frequency of treatment prescribed		BP controlled		BP uncontrolled		Odds ratio	95% CI	*p*
	*n*	%	*n*	%	*n*	%			
HCTZ	484	92.54	212	43.80	272	56.20	1.01	0.52–1.95	0.980
Amlodipine	230	43.98	90	39.13	140	60.87	0.71	0.51–1.01	0.058
Enalapril	341	65.20	135	39.59	206	60.41	0.61	0.43–0.88	0.008*
Atenolol/carvedilol	18	3.44	8	44.44	10	55.56	1.03	0.39–2.64	0.954
HCTZ + Enalapril	174	33.20	70	40.23	104	59.77	0.80	0.56–1.16	0.247
HCTZ + amlodipine	67	12.80	26	38.80	41	61.20	1.07	0.68–1.69	0.767
Single antihypertensive	124	23.70	71	57.30	53	42.70	0.19	0.05–0.33	-
Drug class combinations	399	76.29	158	39.60	241	60.40	0.48	0.33–0.74	0.001*
Lifestyle modification	521	99.62	228	43.93	293	56.24	0.96	0.89–1.03	0.740

Note: Furosemide, carvedilol and methyldopa were excluded because a few of these antihypertensives were prescribed.

BP, blood pressure; HCTZ, hydrochlorothiazide; 95% CI, confidence interval.

* , *p* < 0.05.

## Discussion

### Guideline implementation on documented screening for target organ damage

The study found poor compliance with various aspects of the SAHPG concerning the documentation of demographic information, anthropometry and screening for TOD. Demographic factors including age, race, employment and education are known to influence BP control^30^ and the decision on which antihypertensive therapies to prescribe. Lowering BMI, thereby losing weight, can reduce BP levels, thus improving BP control.^16,31^ In this study, BMI was documented in just over half the medical records, which reflects a gap in the day-to-day practice in primary health care. According to the guidelines, weight should be measured at every visit and height at least once at baseline to be able to calculate BMI at every visit.^11,13,14,16^

A study conducted in Lesotho reported an increase in left ventricular modelling and renal impairment among patients with elevated BP^32^ showing the importance of TOD screening. In this study, screening for TOD was poor as the urine analysis and the ECG tests used to screen for kidney disease and left ventricular remodelling, respectively, were insufficient. Although the urine dipstick, potassium and random blood glucose test were not carried out frequently, these screening tests were performed significantly more often in the diabetes mellitus and dyslipidaemia groups than in the medical records of patients with other comorbidities. The screening tests that were regularly documented included random cholesterol and serum creatinine measurements. Omissions in screening for TOD may have serious implications for hypertensive patients with unidentified coexisting comorbidities.^33^ Another study found that, among other challenges, overwhelming patient numbers seen in primary care facilities compromise the quality of care.^34^

### Recorded blood pressure control in the medical records of hypertensive patients

In this study, the proportion of medical records with BP control was suboptimal with target BP documented in less than half the reviewed files. Similarly, studies in other provinces of SA, such as the Mpumalanga and Gauteng provinces, also found that overall BP control was below target.^20,35^ On the other hand, there are studies that have reported BP control achieved in more than 50% of participants,^36,37^ meaning that there is the potential to achieve better levels of BP control. However, some South African studies have reported much lower levels of BP control than this study.^38,39^ Suboptimal BP control is a reason for concern because of the known increased risks for all-cause and CVD mortality.^40^ The risk of cardiovascular events, strokes and kidney disease is closely related to increased levels of BP. These complications may, therefore, lead to an increase in the burden of disease and economic costs.^41^

### Comorbidities in a cohort of hypertensive medical records: Blood pressure control and management

The three most common comorbid conditions in our study were HIV, dyslipidaemia and diabetes mellitus (Table 3). The proportion of files indicating patients as being HIV positive was unexpectedly high, with levels above numbers nationally recorded and per district.^42,43^ Notably in this study, BP control in the HIV group was significantly better than in the other groups of hypertension comorbidities. In the majority of records of the HIV group, the documented age was above 50 years, likely the result of freely available antiretroviral therapy in SA.^44^ A study by Lebina et al. found a 30% prevalence of hypertension in a virally suppressed cohort of HIV patients above the age of 40 years.^45^ This is consistent with increasing hypertension prevalence with age.^46^ In addition, the group with comorbid HIV was more likely to have a controlled BP, compared to groups with other comorbid conditions. This could be because clinicians were more likely to implement HIV guideline recommendations in this cohort of hypertensive patients. Under the South African HIV treatment programme, where regular annual monitoring is supported by audits, it is a requirement that BP is controlled in a cohort of comorbid HIV patients before referral to a facility-based or external repeat prescription pick-up point.^47^

### Recorded clinician management of hypertension and blood pressure control

Lifestyle advice forms an integral part of the management of patients with hypertension, and it is recommended as a first-line therapy by several guidelines.^11,13,16^ Studies show that high BP can be lowered by maintaining a healthy lifestyle.^16,31^ In this study, lifestyle advice was documented as offered in almost all the medical records reviewed. However, there was no mention of whether the lifestyle advice offered was tailored to the needs of the patient.

All the medical records had at least one documented antihypertensive therapy prescribed, but the proportion of files with controlled BP remained suboptimal. This finding is similar to a study carried out in Gauteng that found BP control low, despite the appropriate antihypertensive classes prescribed.^36^ A thiazide diuretic was the most frequently prescribed antihypertensive agent in the study, in line with the guidelines and indicating it as the agent of choice secondary to lifestyle advice.^11,12,13,14^ The ACEI was the second most prescribed antihypertensive agent, which although appropriately prescribed as a second line in this study, had reduced odds of achieving a controlled BP. Another study found that when a CCB was prescribed with either a thiazide diuretic or an ACEI, the combination of a CCB and thiazide diuretic resulted in better BP control than with a thiazide diuretic and an ACEI.^47^ In addition, our study also found significantly reduced odds of having BP controlled when the number of antihypertensive classes prescribed was increased to more than two antihypertensives. Studies support the use of a single drug fixed-dose combination of antihypertensive for better tolerance,^48^ which is not yet available in the public sector in SA.

### Strengths and limitations

This was a review of patient records and therefore prone to information bias. Because of the study reviewing active patient files, the ethical requirement meant that only the medical records of patients who signed an informed consent were reviewed. The study is therefore prone to selection bias. In addition, the information regarding race, marital status and educational level was not documented in the demographic information section of the patient files and thus the association between BP control and demographic profiles were not assessed. Furthermore, the association between BMI and BP control could not be assessed in the study because of the infrequent recording of height and calculation of BMI. The poor documentation of screening for target organ complications may be interpreted as screening that was not performed. Furthermore, as medical records were reviewed in CHCs located at the peripheries of the town, the sample may not be representative of the South African population. The draining areas served by the CHCs are limited geographically, being not representative of the entire Matlosana Sub-district. Despite the above limitations, this study provides insight into the quality of hypertension management in the sub-district and the findings provide an opportunity for quality improvement projects and continuous medical education on hypertension guidelines.

## Conclusion and recommendations

This study found that documentation regarding the implementation of the SAHPG recommendations among patients with hypertension in the study setting was poor and BP control was suboptimal. The most common documented comorbid illness was HIV. Where HIV was confirmed, better BP control than in other groups of comorbidities was noted. Screening for TOD was generally poorly documented in this study. Healthcare workers need to be aware of the finding of poor documentation of SAHPG recommendations and suboptimal control of BP in this study setting. Programmes that audit and improve the quality of hypertension guideline implementation and BP control in primary care require ongoing support and further research.
